# The companion dog as a model for human aging and mortality

**DOI:** 10.1111/acel.12737

**Published:** 2018-02-19

**Authors:** Jessica M. Hoffman, Kate E. Creevy, Alexander Franks, Dan G. O'Neill, Daniel E. L. Promislow

**Affiliations:** ^1^ Department of Biology University of Alabama at Birmingham Birmingham AL USA; ^2^ College of Veterinary Medicine & Biomedical Sciences Texas A&M University College Station TX USA; ^3^ Department of Statistics University of Washington Seattle WA USA; ^4^ Pathobiology and Population Health The Royal Veterinary College Hatfield UK; ^5^ Department of Pathology University of Washington Seattle WA USA; ^6^ Department of Biology University of Washington Seattle WA USA; ^7^ Present address: Department of Statistics and Applied Probability University of California Santa Barbara Santa Barbara CA USA

**Keywords:** aging, cause of death, comorbidity, companion dog, human, mortality

## Abstract

Around the world, human populations have experienced large increases in average lifespan over the last 150 years, and while individuals are living longer, they are spending more years of life with multiple chronic morbidities. Researchers have used numerous laboratory animal models to understand the biological and environmental factors that influence aging, morbidity, and longevity. However, the most commonly studied animal species, laboratory mice and rats, do not experience environmental conditions similar to those to which humans are exposed, nor do we often diagnose them with many of the naturally occurring pathologies seen in humans. Recently, the companion dog has been proposed as a powerful model to better understand the genetic and environmental determinants of morbidity and mortality in humans. However, it is not known to what extent the age‐related dynamics of morbidity, comorbidity, and mortality are shared between humans and dogs. Here, we present the first large‐scale comparison of human and canine patterns of age‐specific morbidity and mortality. We find that many chronic conditions that commonly occur in human populations (obesity, arthritis, hypothyroidism, and diabetes), and which are associated with comorbidities, are also associated with similarly high levels of comorbidity in companion dogs. We also find significant similarities in the effect of age on disease risk in humans and dogs, with neoplastic, congenital, and metabolic causes of death showing similar age trajectories between the two species. Overall, our study suggests that the companion dog may be an ideal translational model to study the many complex facets of human morbidity and mortality.

## 1 INTRODUCTION

Age is the greatest risk factor not only for the probability of death, but also for the majority of morbidities associated with mortality (Finkel, 2005; Kaeberlein, Rabinovitch & Martin, 2015; Kennedy et al., 2014). However, studies to identify factors that alter patterns of aging using animal models have focused on lifespan and age‐specific mortality, rather than the underlying patterns of morbidity that lead to death. This gap is due in part to the difficulty of measuring causes of mortality in the standard animal models in aging studies. For example, age‐related morbidity and causes of mortality in the commonly studied models of aging range from the not well understood (and often not studied) in mice (Simms & Berg, 1957; Snyder, Ward & Treuting, 2016), to the poorly understood in flies and worms (but see Herndon et al., 2002; Rera, Clark & Walker, 2012), to nonexistent in yeast. In addition, many diseases important to human aging (e.g., cardiovascular disease and dementia) do not develop spontaneously in our commonly studied model organisms. To this end, we need a model organism that allows us to understand not only age‐related mortality, but also age‐related morbidity and causes of death. The companion dog (i.e., dogs that reside under their owner's care) has the potential to fill this gap and to enable us to better understand the genetic and environmental factors that affect lifespan, and the underlying forces that shape age‐specific morbidity and mortality.

Over the last 200 years, individual dog breeds have been highly inbred, with the result that genetic variation is relatively limited *within* breeds, but considerable *among* breeds (Leroy, Verrier, Meriaux & Rognon, 2009; Ostrander, Wayne, Freedman & Davis, 2017; Sutter et al., 2004). Thanks in large part to this history of intense breeding for specific morphological and behavioral traits, dogs are the most phenotypically diverse mammalian species on the planet. This diversity is found not only in morphology and behavior, but also in life‐history traits, where across breeds, dogs exhibit an almost twofold difference in average longevity (Bonnett & Egenvall, 2010; Fleming, Creevy & Promislow, 2011), and enormous variation in risk of specific diseases (Fleming et al., 2011). In addition, companion dogs live in diverse environments and often in close proximity with their owners. A tightly controlled environment in the laboratory may be ideal for some scientific questions, but because we share our environment closely with dogs, they offer us the opportunity to assess how environment influences morbidity and mortality in dogs as well as our own species.

Dogs also have a sophisticated veterinary healthcare system, second only to that of humans, allowing clinicians to diagnose and treat specific diseases, and to identify exact causes of death (American Veterinary Association). For example, unlike mice, companion dogs experience a diversity of spontaneously occurring diseases similar to those of humans, such as age‐related neurologic disease (Head, 2011), renal disease (Cianciolo, Benali & Aresu, 2016), endocrine disease (e.g., De Bruin et al., 2009; Fall, Hamlin, Hedhammar, Kampe & Egenvall, 2007), and also experience obesity and its attendant risks (German, Ryan, German, Wood & Trayhurn, 2010). These phenomena allow researchers not only to study the pathologies that influence mortality, but also to understand different comorbidities and multiple chronic conditions that canines exhibit. In previous work, we showed that multimorbidity in the dog increases with age (Jin, Hoffman, Creevy, O'Neill & Promislow, 2016), similar to patterns seen in humans. As we discuss here, the domestic dog could be a useful model to understand how comorbidities, both as cause and consequence, are associated with aging in humans.

Surprisingly, while we know a great deal about the age‐specificity of human morbidity, substantially less is known about the degree to which other species, including dogs, show similar disease‐specific patterns of aging. Such comparisons are critical in our efforts to develop powerful models to identify the genetic and environmental determinants of morbidity and mortality. Here, we present a comparative analysis of causes of mortality in both humans and companion dogs. We determine the extent to which the companion dog may provide an excellent model of human aging and the degree to which causes of mortality correlate between the two species. Our results lay the groundwork for future use of the domestic dog as a model of human aging and longevity.

## 2 RESULTS

We obtained morbidity and mortality data from 112,375 humans from the U.S. Census Bureau's National Longitudinal Mortality Study, 73,835 dogs in the Veterinary Medical Database (VMDB, 37,480 of which remained after removal of individuals with “unclassified” pathophysiological process [PP] or organ system [OS] and were used for comorbidity PP/OS analysis, see Methods), and 5,095 dogs in the VetCompass Programme database in the United Kingdom. Using the Census human data and VMDB canine data, we were able to compare eight PP and nine OS categories between the two species. There were a total of 106 different possible causes of death for the Census dataset, and these were assigned to a total of 38 of 72 possible PP×OS combinations. For the VMDB, 5563 different diagnoses were recorded representing 93 PP×OS combinations. The VetCompass dataset had 56 diagnoses that were not able to be placed into PP and OS cause of death, and which had no information on comorbidity, and as such were only used for longevity analysis.

**Figure 1:**
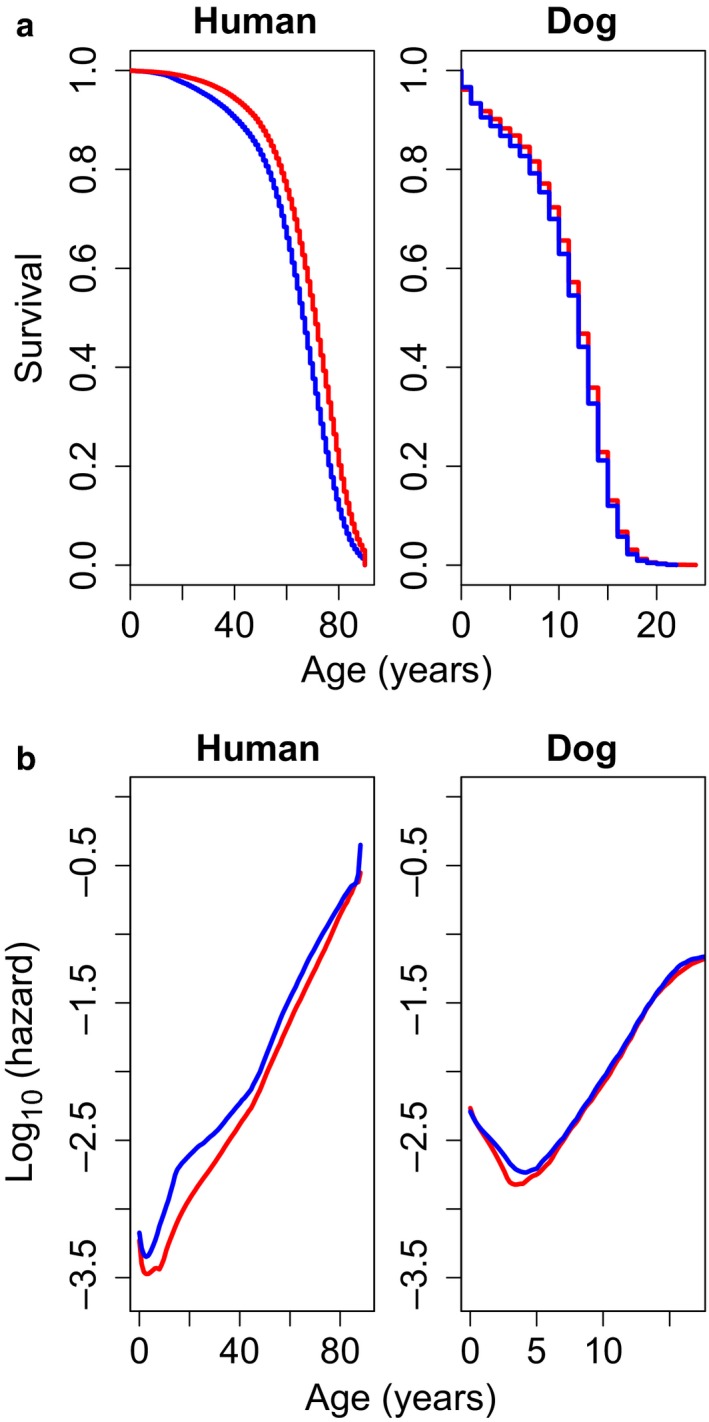
Survivorship (a) and hazard curves for (b) for human (left) and dogs (right). Human data are from the U.S. Census Bureau (1972–2002), and canine data come from the VetCompass database (2010–2013). For both species, colors represent the two sexes, female (red) and male (blue). Gompertz slope parameters were calculated for humans: 0.089 (females) and 0.080 (males), and dogs: 0.0214 (females) and 0.0219 (males)

We first attempted to determine whether survivorship curves in humans and dogs were similar. Overall trends were similar between the two species (Figure 1a), with females being longer lived than males. However, our Cox proportional hazard model showed only a minor effect of sex on longevity in dogs, as reported previously from the data (Hoffman, O'Neill, Creevy & Austad, 2017), while there was a much larger effect in humans (Cox proportional hazard, *p* = .017 for dogs, *p* < .001 for humans). Similar results are seen in our hazard plots (Figure 1b), where human males have increased mortality compared to females at all ages, with no sex effect seen in dogs. The slopes of the hazard curves suggest significant differences in the rates of aging in the two species, especially in the earliest ages, with dogs having higher starting hazards as compared to humans (Figure 1b, Gomperz slopes—humans: 0.089 (females) and 0.080 (males), and dogs: 0.0214 (females) and 0.0219 (males)).

### 2.1 Canine multimorbidity and comorbidity analysis

We first looked at the effect of age and sex on multimorbidity (counts of all diagnoses at time of death from the VDMB data) using a generalized linear model (GLM) with a negative binominal distribution. Our analysis indicated a significant positive association between age and number of morbidities (*p* < .001), but no significant effect of sex on morbidity count (*p* = .97, Figure S1). We then determined how PP and OS categorical causes of death were associated with multimorbidity count (Figures S2 and S3). After controlling for the effects of age, there was no association between PP cause of death and total counts of morbidities. However, OS cause of death did have a significant association with morbidity count, even after controlling for the effects of dog age. In particular, dogs that died of urogenital and hepatic causes had higher numbers of morbidities than dogs dying of other causes (Figure S3). As in our analysis of the effects of sex on multimorbidity number after controlling for age, sex had no significant effect on morbidity number in either the PP or OS model (*p* > .27 for both).

We next investigated the morbidity count associated with each of five specific chronic conditions in dogs (comorbidity analysis) and found that having a diagnosis of any of four conditions—diabetes mellitus, arthritis, obesity, and hypothyroidism—was associated with an increase in comorbidity number (*p* < .0002 for all specific morbidities, Figure S4). Arthritis, obesity, and hypothyroidism show more than double the number of comorbidities as compared to the overall dataset. Dogs diagnosed with chronic kidney disease did not show a significant increase in comorbidities compared to the overall population (*p* = .84). Similar to our multimorbidity analysis (combined number of diagnoses), our comorbidity analysis (number of diagnoses a dog had when one was a morbidity of interest) did not point to a significant effect of sex on number of comorbidities.

### 2.2 Human–dog comparisons

**Figure 2:**
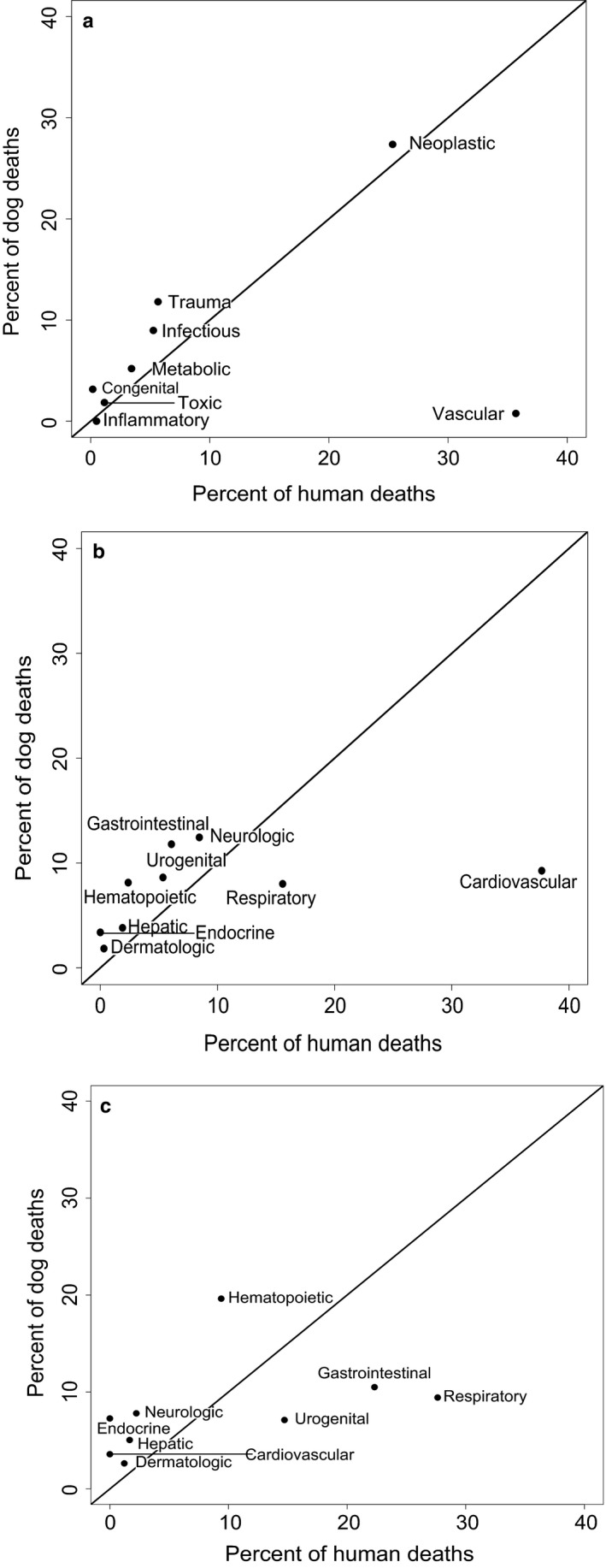
Correlation between pathophysiological process (a), organ system (b), and specific cancer (c) causes of death in humans and dogs in the VMDB database (1984–2004). (a) Correlation *p* = .58 by Spearman rank test, ρ = 0.238. Removing vascular causes of death results in a significant correlation between humans and dogs (ρ = 0.857, *p* = .023). (b) Spearman rank ρ = 0.733, *p* = .031. (c) For those causes of death that had a “neoplastic” process, graph depicts the organ systems in which cancer occurred. Spearman rank ρ = 0.661, *p* = .053

The major objective of this study was to explore shared patterns in causes of death between humans and dogs. Across PP categories, we discovered many PP causes of death that showed similar proportions between dogs and humans. However, the overall correlation between humans and dogs in causes of death was not significant via a Spearman rank test (Spearman ρ = 0.238, *df* = 6, *p* = .58, Figure 2a). The largest difference between the two species is seen with respect to vascular causes of death, which are much more prevalent in the human population. When this category is removed, we see a significant correlation among the remaining seven PP causes (Spearman ρ = 0.857, *df* = 5, *p* = .024).

We found a significant correlation in rates of death due to OS categories in humans and dogs (Spearman ρ = 0.733, *df* = 7, *p* = .031, Figure 2b). While large differences were seen in proportions of respiratory, gastrointestinal, and urogenital causes of death between humans and dogs, the proportion of neoplastic deaths is nearly identical between the two species (25.3% in humans; 27.4% in dogs; Figure 2c), although cancer risk is generally greater in large‐breed than small‐breed dogs (Fleming et al., 2011). Despite the similarity in risk of death due to neoplasia among humans and canines, the types of cancer seen in each species are only marginally correlated (Spearman rank ρ = 0.661, *df* = 7, *p* = .053).

**Figure 3:**
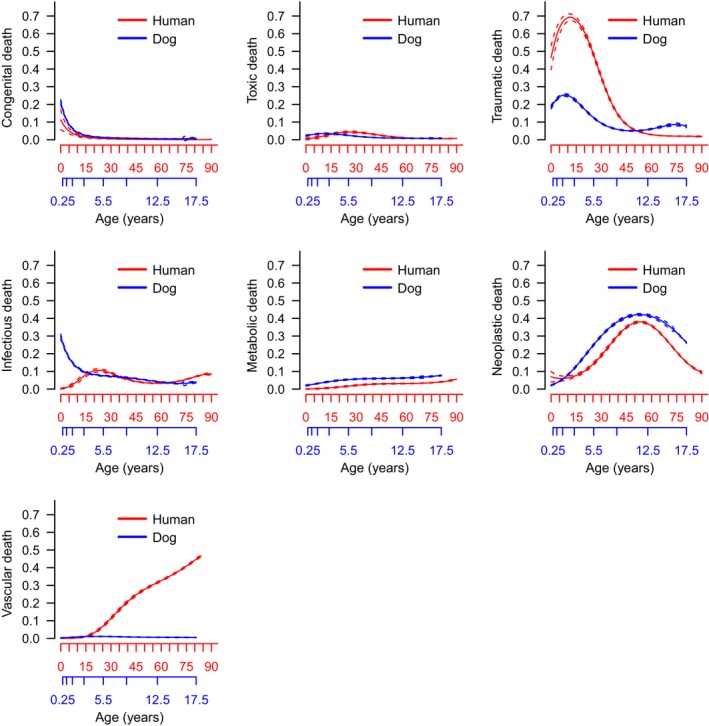
Proportions of human and dog pathophysiological process causes of death with age. Humans are represented in red with dogs in blue. Dashes indicate 95% confidence intervals

After comparing overall causes of death between the two species, we determined how age trajectories of different causes of death compared between the two species. For many PP categories, very similar age trajectories are seen, especially for neoplastic, congenital, toxic, and metabolic causes of death (Figure 3). For traumatic death, mortality in humans was biased toward young adults, a pattern also observed in dogs. Our odds ratio analysis suggests age trajectories for risk of both metabolic and neoplastic deaths are similar between dogs and humans except at the earliest ages (when deaths are rare), while vascular diseases are much more common in humans compared to dogs (Figure S5). Controlling for age, risk of cancer is most similar between humans and dogs for a human age of 53 years and a dog age of 11.5 (odds ratio close to zero). On the other hand, the odds that a human death at age 75 has a vascular cause is over 2^6^ = 32 times larger than it is for dogs at the appropriately scaled age (65 human years), with a log odds ratio greater than 6 (Figure S5).

**Figure 4:**
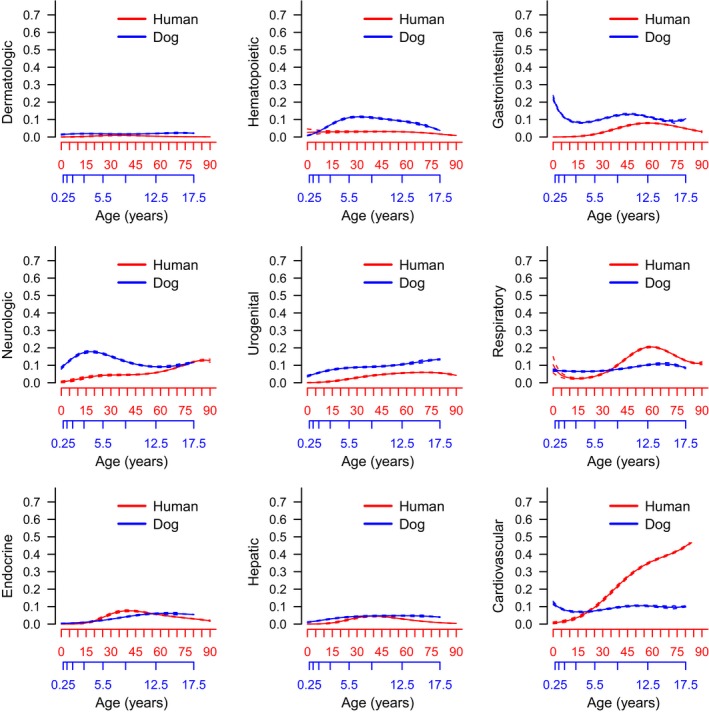
Proportions of human and dog organ system causes of death with age. Humans are represented in red with dogs in blue. Dog years have been rescaled to approximate human years as described in Methods. Dashes indicate 95% confidence intervals

We next compared human and canine age trajectories for OS categorical causes of death. Endocrine and hepatic causes of death show very similar age trajectories, while many of the other OS causes of death do not (Figure 4). The only major outlier is the age trajectory for cardiovascular categorical causes of death, which is different both in shape (increasing throughout life in humans, steady in dogs) and magnitude (higher percentage in humans). The log odds ratio plots demonstrate that cardiovascular causes of death are as much as 2^3^ = eight times higher in humans at late age while hepatic and metabolic causes of death are more common among dogs at late age (Figure S6).

**Figure 5:**
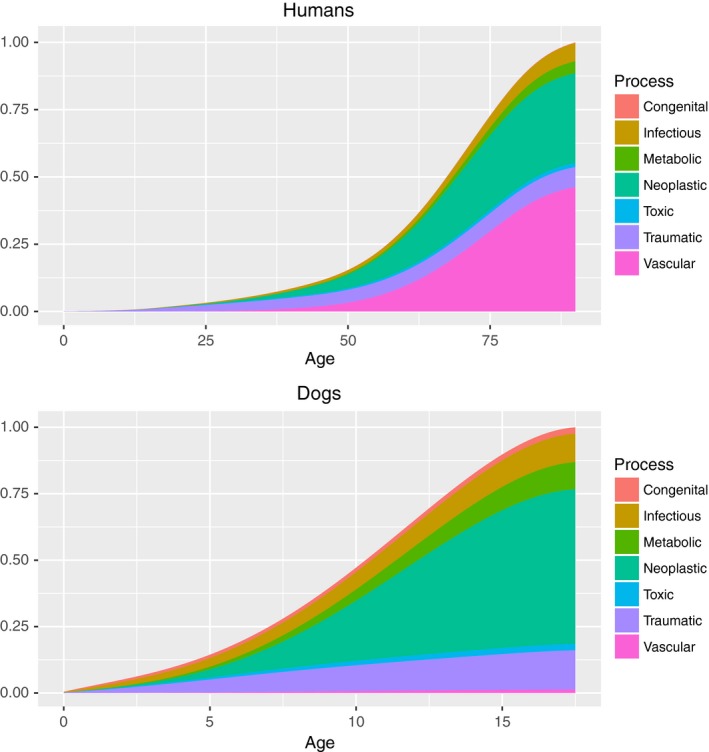
Stacked density plots of pathophysiological process causes of death with age for humans (top) and dogs (bottom). Congenital causes of death in humans were too small to be visible in the figure

**Figure 6:**
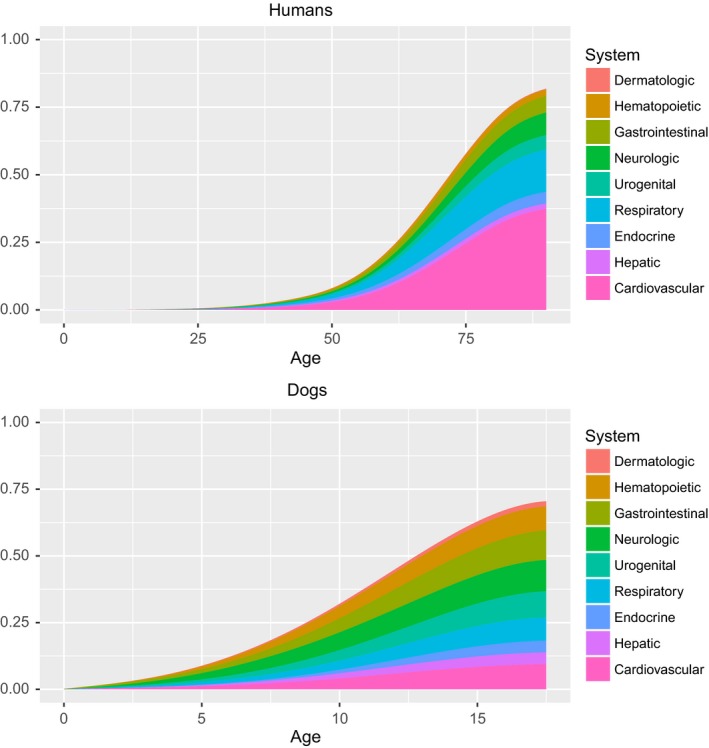
Stacked density plots of organ system causes of death with age for humans (top) and dogs (bottom)

In addition to frequencies of overall deaths at each age, we also determined which causes of death were age related in the two species (absolute number of deaths increase with age) by plotting density plots across the lifespan for all process (Figure 5) and system (Figure 6) causes of death. For both species, the majority of PP causes of death were age related with the exception of congenital and traumatic categories in both species, and infectious and toxic categories in dogs only. In both species, all OS causes of death showed increasing numbers with age.

## 3 DISCUSSION

Many authors have pointed out that age is the single greatest risk factor for a variety of causes of death (Finkel, 2005; Kaeberlein et al., 2015; Kennedy et al., 2014). However, with few exceptions, we know relatively little about the factors that determine how age shapes disease risk, why rates of aging (i.e., age‐specific rates of increase) differ among diseases, and why some diseases show relatively few signs of aging at all. In some cases, most notably cancer, we have been able to develop mathematical models that are consistent with age trajectories (Armitage & Doll, 1954; Frank, 2007).

In both humans and dogs, cancer is a leading cause of death, and age trajectories of cancer deaths are almost identical between the two species (Figure 3). This suggests that, as some previous studies have suggested, the domestic dog is an ideal model for understanding human cancer (Marconato, Gelain & Comazzi, 2013; Rowell, McCarthy & Alvarez, 2011). However, when we analyzed the specific types of neoplasia associated with death in the two species, we found significant differences. Respiratory, urogenital, and gastrointestinal cancers were more common causes of death in humans compared to dogs. These three particular cancers are highly associated with lifestyle factors, including smoking, obesity, and diet. Thus, if we could reduce the number of human cancer deaths due to lifestyle factors, we might see a shift in the overall distribution of different types of cancer in humans to one more closely resembling that in dogs.

Similar age‐specific trajectories were also seen between the two species with respect to congenital and metabolic causes of death. Given that congenital causes of death affect early‐age mortality, it makes intuitive sense that the overall age distribution is similar in both species. Similarities in the age distribution of the metabolic causes of death were also notable. Metabolic causes of death are often associated with lifestyle factors, especially obesity, and are projected to increase in prevalence as rates of obesity increase in humans in the United States (Flegal, Carroll, Kit & Ogden, 2012). Interestingly, in our comorbidity analysis (number of diagnoses recorded for dogs with a specific morbidity of interest), the diagnosis of obesity in dogs was associated with more than double the number of comorbidities as the overall population. This is similar to results seen in humans, in which obesity is associated with more chronic conditions than smoking or excessive drinking (Sturm, 2002), and previous research has shown that obese humans are more likely to own obese dogs (Nijland, Stam & Seidell, 2010). However, it should be noted that because of the varied morphology of dogs, a reliable BMI metric has proved elusive, and a semi‐quantitative body condition score (BCS) is customarily used by veterinarians. As such, while obesity diagnoses in the VMDB were assigned by attending veterinarians, there is not a numeric BMI which can be referenced for each of these diagnoses. Combined, the dog is well positioned to be an excellent model of obesity and metabolic disorders.

While many causes of death in dogs and humans share similar relative age distributions, cardiovascular disease stands out as being strikingly different between the two species. The reduced prevalence of heart disease as a cause of death in canines is interesting considering dogs share many environmental influences with humans. A large proportion of companion dogs in the United States are obese (Freeman et al., 2006), yet dogs are rarely diagnosed with myocardial infarction. This is due at least in part to dogs having blood cholesterol profiles associated with a low risk of cardiovascular disease with elevated high‐density lipoprotein and reduced low‐density lipoprotein profiles (Tsutsumi, Hagi & Inoue, 2001). As a result, dogs do not develop atherosclerosis except in rare cases when a concurrent condition causes a dramatic increase in total cholesterol levels (e.g., diabetes and hypothyroidism) (Hess, Kass & Van Winkle, 2003). Given the lack of similarity between dogs and humans in patterns of cardiovascular disease and its attendant risks, some might argue that the dog is a less than ideal model for human vascular diseases. At the same time, this stark difference in risk despite similar environments suggests that close genetic and physiological analysis of the differences between humans and dogs could shed light on mechanisms that enhance species resilience against myocardial infarctions, even in the presence of specific risk factors.

As expected, we found that most causes of death in both species were highly age related, even for those disorders whose proportional contribution to overall deaths declined at late age compared to earlier ages. Both species show a decline with age in proportions of congenital and traumatic causes of death. While the curves are similar, total numbers of traumatic death are quite different, with many more canines presenting with traumatic deaths. Interestingly, while infectious disease causes of death are age related in human populations, this is not the case in the canine population, in which numbers of individuals dying of infectious diseases do not increase with age. In a similar vein, inflammatory causes of death peak early in the life course of the dog while showing increases in later ages in humans. Different inflammatory and immune responses exist between the dog and human, although some studies point to similarities in age‐related patterns of immune markers. Previous research has shown age‐related declines in immune markers in companion dogs (Greeley, Kealy, Ballam, Lawler & Segre, 1996; Greeley et al., 2001), as well as age‐associated changes in inflammatory markers (Alexander, Colyer, Haydock, Hayek & Park, 2017), similar to humans. Future studies are necessary to understand why many similar changes in immune and inflammatory response with age are seen, yet differences in mortality between the two species are observed.

In addition to direct causes of death, the co‐existence of multiple chronic conditions within individual patients has been a large focus in aging research due to the increasing prevalence of multimorbidity in developed nations (Wolff, Starfield & Anderson, 2002), and evidence that increased numbers of chronic conditions are associated with decreased life expectancy (DuGoff, Canudas‐Romo, Buttorff, Leff & Anderson, 2014). Our multimorbidity analysis suggests that companion dogs have the potential to be an excellent model to better understand the causes and consequences of multiple chronic conditions as we age. Similar to previous results reported by our laboratory (Jin et al., 2016), multimorbidity (total number of diagnoses recorded at the time of death) increases with age in the dog as is seen in human populations (Fortin, Bravo, Hudon, Vanasse & Lapointe, 2005). In addition, comorbidities vary significantly among different chronic conditions in the dog (Figure 3). It should be noted, however, that we found no significant effects of sex on multimorbidity numbers, which is in line with previous results suggesting no sex difference in longevity nor cause of death in the dog (Hoffman et al., 2017). This contrasts with results seen in humans, where women tend to have higher rates of multimorbidity than men (Agur, McLean, Hunt, Guthrie & Mercer, 2016).

The similarity between our canine analysis and previous human studies with respect to patterns of comorbidity suggests the companion dog may provide an ideal model to study multimorbidity (multiple chronic conditions) and comorbidity (additional diagnoses concurrent with a specific diagnosis of interest) in a way far superior to other model organisms. The companion dog's significantly shorter lifespan, median of 12 years (O'Neill, Church, McGreevy, Thomson & Brodbelt, 2013), than that of humans could allow us to understand the consequences of multiple chronic conditions in longitudinal studies lasting just a few years instead of the decades needed for human studies.

Finally, in addition to the translational value of many aspects of canine aging for human medicine, and the obvious direct benefit of improved healthspan to the dogs themselves, research on canines has the potential to provide diverse benefits to human quality of life. Dogs are utilized in many working capacities including service dogs, police dogs, and livestock guard dogs. Training these dogs often takes an investment of many months to years, plus thousands of dollars (National Police Dog Foundation; Wirth & Rein, 2008), and improving their healthspan could give humans more working years with each dog. In addition to working roles, dogs can reduce stress and improve emotional well‐being in their owners, increasing human quality of life (Friedmann & Son, 2009; Ownby, Johnson & Peterson, 2002; Virues‐Ortega & Buela‐Casal, 2006; Walsh, 2009). Thus, identifying ways to improve canine healthspan could not only have a translational impact on humans, but could also have significant direct economic and personal benefits for people.

### 3.1 Caveats

While our analyses suggest that companion dogs hold out great promise as a model of human aging and disease, there are several limitations to this study. First, much of our canine data may reflect only a subset of the general canine population. The VMDB data consist of data from dogs that were seen at veterinary teaching hospitals. Patients at these tertiary care facilities are more likely to present with cancers and rare or serious diseases (Bartlett, Van Buren, Neterer & Zhou, 2010). Also, the VMDB age data are collapsed into bins. We might know that a particular dog died somewhere between the ages of 10 and 15, for example, but not its exact age. While we can fit parametric models to these data using interval‐censored models (e.g., Kraus, Pavard & Promislow, 2013), there is considerable error in these estimates. Also, the VMDB data include only the diagnoses given to each dog and do not specify the diagnostic path that was pursued to achieve the diagnoses. Thus, we cannot know, for example, whether an infectious disease diagnosis was reached by serologic or molecular testing, and whether a neurologic diagnosis was achieved by clinical signs, diagnostic imaging, histopathology, or a combination of those. The VetCompass dataset provides more accurate age information, but does not have detailed cause of death information nor does it capture all comorbid diagnoses that each dog had at the time of death.

In addition, neither dataset includes information about previous diseases or the environments in which the dogs resided. This includes the diets the dogs were fed by their owners. Commercial pet diets make up over 90% of all food fed to companion dogs, but these vary greatly in nutritional profiles, similar to diets in humans. As it would be expected that diet will have a significant contribution to the development of morbidities in the dog just as seen in humans, future prospective studies are needed to ascertain the extent to which individual diets affect morbidity and lifespan in the dog. Finally, our canine analysis makes the assumption that all dogs are drawn from the same pool and does not look at the effects of breed and size variation. Previous research has shown that even within a size class, breeds can differ in their multimorbidity (Jin et al., 2016) and mortality patterns (Fleming et al., 2011). Taken together, these limitations point to the need for more detailed data from canines representing the full diversity of ages, breeds, comorbidities, and environments. Ideally, data from a prospective longitudinal study of aging in dogs would allow us to more accurately determine how morbidity and mortality correlate between dogs and humans (Kaeberlein, Creevy & Promislow, 2016), and the role of multiple morbidities, both as cause and consequence, in the aging process.

We could not ascertain comorbidities from the U.S. Census human data. For more accurate comparisons between humans and dogs, all environmental conditions and morbidities throughout life need to be studied. Currently, many human questions are being answered by long‐term longitudinal studies (Shock et al., 1984), and a dog longitudinal study would nicely complement these studies already being completed in humans. While these human studies have been ongoing for decades, a canine longitudinal study could be completed before human studies end due to the shorter lifespans of the animals (<20 years).

Finally, one detriment to working with the dog as a model for human health is that our companion animals are often euthanized when their quality of life becomes low. In the UK, 86.4% of dogs are recorded as dying by euthanasia (O'Neill et al., 2013). This can make it difficult to ascertain an actual cause of death as owners may elect to euthanize a dog who could conceivably have survived the current diagnosis and experienced a later, alternate cause of death. This is a consideration to include with any companion animal model system as it introduces some bias into determining actual causes of death. Paradoxically, this may also improve the usefulness of canine data for aging studies, especially those with measures of healthspan, defined as the number of healthy years of life. Many euthanasia decisions are based on the owner's perception that the quality of life (both present and future) has dipped below an acceptable level and therefore the medical causes for euthanasia decisions may be very useful metrics for healthspan. Conversely, in humans, the drive to life extension means that many persons will have remedial and palliative management of initial severe conditions such that they die later of secondary but noninciting causes. Therefore, the cause of death data may not truly describe the most important morbidities that end an individual's healthspan but may instead feature the terminal events that led to the death. Euthanasia in dogs provides a potential endpoint of healthspan such that we can discern those morbidities that are associated with the end of an individual's healthy lifespan.

## 4 CONCLUSIONS

Here, we have presented a large‐scale comparison of human and companion dog mortality. The study findings suggest the dog could be an excellent model to study diverse causes of morbidity and mortality that also affect humans. However, many data are still lacking. A long‐term longitudinal study of aging in domestic dogs, representing a diversity of genotypes and environments, would allow for a more accurate understanding of the promises and pitfalls of the companion dog as a model of human morbidity and mortality.

## 5 EXPERIMENTAL PROCEDURES

### 5.1 Human morbidity and mortality data

We obtained human mortality data from the U.S. Census Bureau's National Longitudinal Mortality Study, which was carried out over a 20‐year period starting in the early 1970s (1973–2002, U.S. Census Bureau). All individuals were followed for 10 years (in two separate cohorts), and for those who died during this period, age and cause of death were recorded. These causes of death were then assigned to a specific pathophysiological process (PP) and organ system (OS) as described previously in Fleming et al. (2011), such that each individual had both a process and system classification of death. Pathophysiological process describes the mechanism leading to the disease and includes congenital, degenerative, infectious, inflammatory (encompassing immune‐mediated), metabolic, neoplastic, toxic, traumatic, and vascular. Organ system designates the primary organ system that was affected and includes cardiovascular, dermatologic, endocrine, gastrointestinal, hematopoietic, hepatic, musculoskeletal, neurologic, ophthalmologic, respiratory, and urogenital. The option of “unclassified” existed for both pathophysiological process and organ system, for those diagnoses too general or vague to be assigned with certainty. Human causes of death were assigned OS and PP classifications identical to those used for the canine dataset (described below). For rare causes of death that were novel to the human dataset, one of the authors (KEC) assigned a PP and OS in the same manner.

Canine data were collected from two sources. First, we obtained data on cause of death and multimorbidity for dogs from the Veterinary Medical Database (VMDB, https://vmdb.org),^1^ which contains abstracted medical record information submitted by the 27 participating Veterinary Teaching Hospitals in North America. Data obtained included all diagnoses recorded by the attending veterinarians at the time of death for all dogs whose death was documented in the VMDB between 1984 and 2004 (*N* = 73,835 dogs; mean number of diagnoses = 2.97; range of 1–32). For dogs with multiple diagnoses, a single cause of death, including process and system, has been assigned previously (Fleming et al., 2011). Age at death in the VMDB was grouped into bins. We combined the three youngest bins (0–2 weeks, 2 weeks‐2 months, and 2–6 months) into one group labeled 0.25 years; for all other age bins, midpoint ages for each bin were used as in Hoffman, Creevy and Promislow (2013). Animals in the “over 15 years” bin were represented with an age of 17.5 years.

We obtained a second set of canine data, including exact age and cause of death, from the VetCompass Programme database, maintained by the Royal Veterinary College in the United Kingdom (O'Neill, Church, McGreevy, Thomson & Brodbelt, 2014). Because the VMDB represents Veterinary Teaching Hospital patients, it is subject to referral bias (Bartlett et al., 2010), meaning these patients likely exhibit more severe, complicated, or unusual diseases that are seen in the canine population at large, and average income of clients might be higher than that for all dog owners. By contrast, the VetCompass dataset is comprised of complete clinical records from primary care veterinary practices, and as such might provide a better representation of the canine population as a whole. While causes of death were recorded for all dogs, data were grouped into a limited set of 56 specific causes, prior to our obtaining the data. Due to overlap in many of the diagnoses, OS and PP classifications could not be completely assigned as was done with Census and VMDB data. Ethical approval for the VetCompass component of the study was granted by the Royal Veterinary College Ethics and Welfare Committee (reference number 2015/1369).

### 5.2 Analyses

All statistical analyses were carried out using the R programming language (R Core Team 2013).

### 5.3 Mortality analyses

We first plotted Kaplan–Meier survival curves for the humans and dogs, keeping the sexes separate. We used a Cox proportional hazard model to determine significant sex effects within each species. We then computed log_10_ hazard mortality values using the muhaz package. We used the flexsurv package to determine parameter values for the Gompertz equation, μ_0_ = αe^βx^, where μ_*x*_ is the instantaneous rate of mortality at age *x*. Gompertz curves are typically plotted on a log‐linear scale: log(μ_*x*_) = log(α) + β*x*. The slope of this line, β, is considered the rate of aging. For this analysis, we included the human data and the VetCompass canine data only, due to the large age bins of the VMDB data.

### 5.4 Canine multimorbidity and comorbidity analyses

While the VMDB does not include information on multiple chronic conditions experienced throughout the lifetime in an individual, we were able to explore the relationships among age and cause of death and number of morbidities at time of death. For this analysis, multimorbidity was defined as the total number of diagnoses recorded at the time of death (Jin et al., 2016), and comorbidity was defined as the number of diagnoses recorded for a dog with a specific morbidity of interest, excluding the specific morbidity of interest. Each diagnosis was weighted equally in our calculation of comorbidity or multimorbidity, regardless of the relative severity of each particular diagnosis. Causes of death that were recorded as “unclassified” in the VMDB were removed, as were all cases in which the only diagnosis was “euthanasia”.

Using the canine VMDB dataset, we first determined how the number of diagnoses changed with age across both sexes using a generalized linear model (GLM), where the dependent variable, multimorbidity, was assigned a negative binomial distribution (Jin et al., 2016). We modeled the impacts of sex and age on morbidity score as fixed effects. Next, we analyzed specific PP and OS categories as described previously (Fleming et al., 2011) to determine whether dogs with certain categorical causes of death exhibited greater multimorbidities than the rest of the population. We used a GLM with pathophysiological process (or organ system), age, and sex as fixed effects as predictors of total number of diagnoses, for which we assumed a negative binomial distribution.

To create a direct comparison between patterns of comorbidity in dogs and humans, we were interested in associations of comorbidities with certain discrete diagnoses that had been entered for individual dogs by their attending veterinarians at the time of the dogs’ death. We looked at comorbidity counts associated with each of five specific diagnoses that are common chronic conditions in both the dog and the human: diabetes mellitus, arthritis, obesity, hypothyroidism, and chronic kidney disease. We used these data to determine whether dogs with these particular diagnoses were more likely to have higher comorbidity counts than dogs without these diagnoses. Similar to our multimorbidity analysis, we considered every diagnosis in each individual dog as a morbidity regardless of the seriousness of the condition. For each morbidity of interest, we ran a GLM with a negative binomial distribution, with the morbidity of interest, age, and sex as fixed effects predicting comorbidity number.

### 5.5 Human–dog comparisons

Our final analysis determined similarities of causes of death between humans and dogs. We first looked at the associations between percentage of deaths for all PP and OS categories in the human and the VMDB canine dataset using a Spearman rank correlation analysis.

Next, we compared causes of death as a function of age for the same PP and OS categories for both humans and dogs. For these analyses, for all individuals who died at a specific age, we estimated the proportion who died of each specific PP/OS. We modeled each cause of death as a function of age using a multinomial logistic regression. For each species (dog and human) and cause of death, we modeled the log odds of death in each group as a fourth‐order polynomial in age:

(1) $$p_{\mathrm{group}(\mathrm{age})}\;=\;\text{softmax}\left(\alpha_{\mathrm{group}}+\sum_{i=1}^{4}\beta_{\mathrm{group}}^{i}{\text{age}}^{i}\right)$$

where $\text{softmax}{\left(z\right)}_{i}\;=\;\exp\left(z_{i}\right)/\left(\sum_{j}\exp(z_{j})\right)$. To facilitate direct comparison, we rescaled dog age to human age by aligning the neoplastic death curves in humans and dogs. Specifically, to align the curves, we found the age at which the proportion of neoplastic deaths is the highest for both humans (53 years) and dogs (11.5 years). This led to one dog year corresponding to approximately 53/11.5 = 4.6 human years. Note that this was derived from analysis using data for all dogs. Scaling would differ for specific breeds as lifespan and risk of cancer vary among breeds. This breed‐specific analysis is beyond the scope of the current analysis and will be explored in the future.

We also included an explicit comparison between humans and dogs by computing the log_2_ odds ratio of death for each cause between humans and dogs:

(2) $$\log\_2\;\text{odds ratio}\;=\;\text{logit}\left(p_{\mathrm{human}(\mathrm{age})}\right)-\text{logit}\left(p_{\mathrm{dog}(\mathrm{rescaled}\;\mathrm{age})}\right)$$

where logit(p)=log_2(p/(1−p)). This quantity tells us how the relative risk of death associated with a given cause occurring changes with age. When the log odds ratio is zero for a particular cause at a given age, this tells us that the odds of this cause of death are the same for dogs and humans. When it is positive, this cause of death is more likely in humans, and conversely when it is negative the cause is more likely in dogs. We then colored a few key groups (PP: vascular, metabolic, and neoplastic; OS: cardiovascular, endocrine, and hepatic) and plotted the other groups in light gray. We also plotted absolute percentages of individuals that died of specific causes at each age to discover which causes of death in both sexes were age related.

## AUTHOR'S CONTRIBUTION

JMH and DELP designed the experiment. JMH and AF completed the data analysis and made the figures. KEC divided causes of death in the human dataset. DGO provided and assisted with the VetCompass data. JMH wrote the first draft of the manuscript, and all authors commented and revised the manuscript.

## CONFLICT OF INTEREST

The authors declare no conflict of interests.

## NOTE

1 The VMDB does not make any implicit or implied opinion on the subject of the paper or study.
