# Supplementary material for: The companion dog as a model for human aging and mortality
Source: Aging Cell. 2018 Feb 19;17(3):e12737. doi: 10.1111/acel.12737 (PMC5946068; doi:10.1111/acel.12737)
Supplement: Supplementary file 1 [file ACEL-17-e12737-s001.docx]

**Supplementary Figures**

**Supplementary Figure 1. Plot of multimorbidities in dogs as function of age at death.** Females are in red, males in blue. Error bars indicate +/- one standard error. Morbidities increase significantly with age (p<0.001) with no significant sex differences (p=0.97).

**Supplementary Figure 2. Number of comorbidities as a function of PP cause of death.** Females are in red, males in blue. Each pathophysiological process was the cause of death for the canine. Error bars indicate +/- one SEM.

**Supplementary Figure 3.** **Number of comorbidities as a function of OS cause of death.** Females are in red, males in blue. Each system was the cause of death for the canine. Error bars indicate +/- one SEM.

**Supplementary Figure 4. Mean number of comorbidities in dogs with a specific diagnosis.** Overall represents the entire dataset. Females are in red, males in blue. Error bars indicate +/- one SEM. CKD = chronic kidney disease. All morbidities of interest were significantly different from the population at large (P<0.002 for each morbidity) with the exception of CKD (P=0.84).

** Supplementary Figure 5. Log-odds ratios of pathophysiological process causes of death between humans and dogs across the lifespan.** Negative log-odds values suggest the cause of death is more common in dogs while positive values are more common in humans at that relative point in the lifespan. A value of zero indicates the relative risk of the cause of death is the same between the species. Colored lines indicated interesting pathophysiological processes. Grey lines indicate the other PP processes analyzed in this study.

**Supplementary Figure 6. Log-odds ratios of organ system causes of death between humans and dogs across the lifespan.** Negative log-odds values suggest the cause of death is more common in dogs while positive values are more common in humans at that relative point in the lifespan. A value of zero indicates the relative risk of the cause of death is the same between the species. Colored lines indicated interesting organ systems. Grey lines indicate the other organ systems analyzed in this study.
